# Coexistence of COVID-19, Pseudomonas, and thoracic actinomycosis in a cystic bronchiectasis case

**DOI:** 10.1186/s12879-023-08215-x

**Published:** 2023-04-06

**Authors:** Emine Afsin, Furkan Kucuk, Hüsna Ozcelik, Muhammed Yavuz Haktanır

**Affiliations:** 1 Department of Chest Diseases, Abant Izzet Baysal University Hospital, Golkoy Bolu, 14200 Turkey; 2 Department of Pathology, Abant Izzet Baysal University Hospital, Golkoy Bolu, 14200 Turkey; 3 Department of Thoracic Surgery, Abant Izzet Baysal University Hospital, Golkoy Bolu, 14200 Turkey

**Keywords:** COVID-19, *Pseudomonas aeruginosa*, Thoracic Actinomycosis, Cystic bronchiectasis

## Abstract

Actinomycosis often leads to cervicofacial infections, but thoracic involvement may also occur. However, the development of empyema is rare. While being followed up with the diagnosis of asthma and bronchiectasis, our case was hospitalized for infected bronchiectasis. As empyema developed in the follow-up, the pleural effusion was drained by tube thoracostomy. Actinomycosis was diagnosed through pleural effusion cytology. Growth of Pseudomonas aeruginosa was observed in sputum culture, and SARS-CoV2 RT-PCR was also positive in nasopharyngeal sampling. Polymicrobial agents can often be detected in actinomycosis. Actinomycosis cases have also been reported in the post-COVID period. Our case is presented since it would be the first in the literature regarding the coexistence of COVID-19, Pseudomonas, and thoracic Actinomycosis (empyema).

## Background

Microorganisms of the genus Actinomyces are gram-positive, anaerobic, non-acid-resistant bacillus determined in the oral flora and cause granulomatous and suppurative infections. While they usually cause cervicofacial infections, thoracic involvement with a rate of 15% may also occur, affecting the lung parenchyma, central airways, pleura, mediastinum, and chest wall [1]. Our case is presented since the coexistence of Coronavirus Disease-19 (COVID-19), Pseudomonas, and thoracic Actinomycosis (empyema) in Actinomyces infections, in which polymicrobial agents are frequently detected, has never been reported before.

## Case presentation

**Fig. 1:**
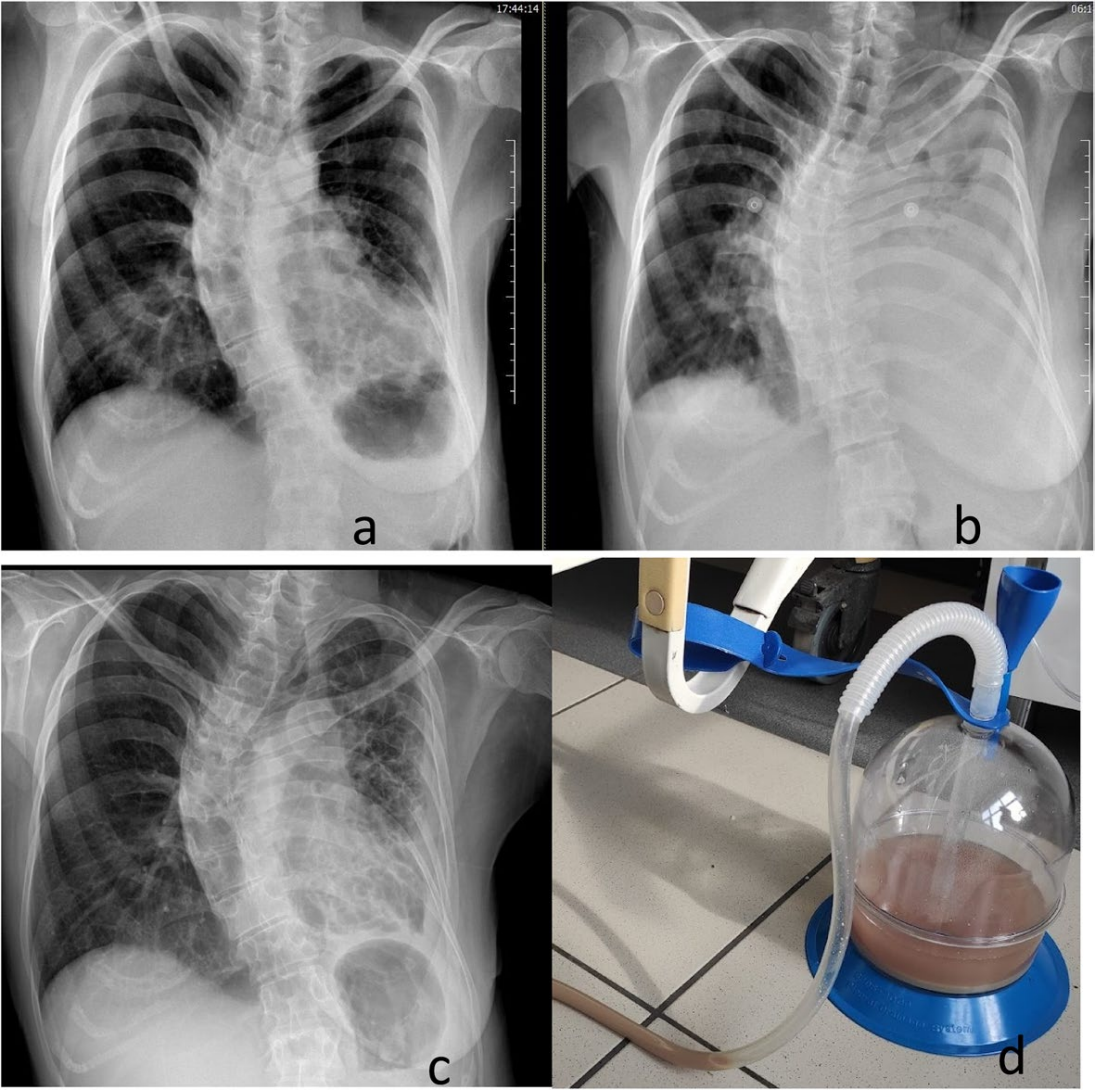
**a** Chest X-ray at hospitalization; **b** chest X-ray when empyema was detected; **c** chest X-ray at the second month of treatment; 1**d** Empyema drainage with chest tube

**Fig. 2:**
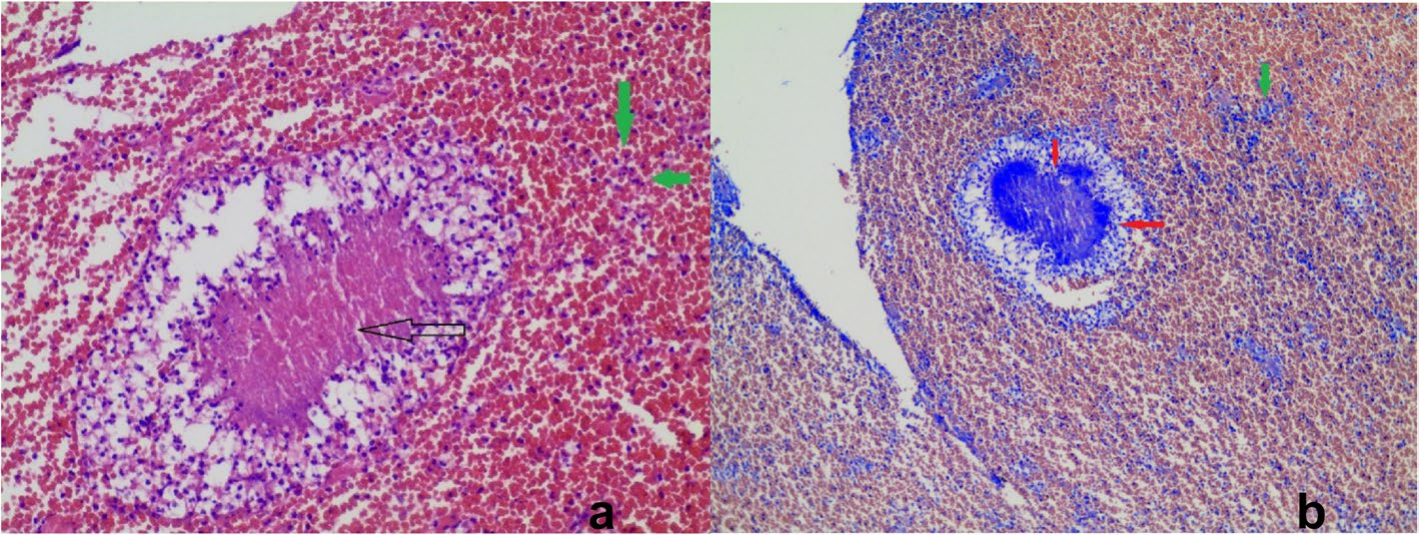
Cell block sections **a** H&E staining; the black arrow indicates sulfur granules and the green arrow inflammatory cells **b** Giemsa staining; green arrow indicates inflammatory cells and the red arrow filamentous bacteria

A 49-year-old female patient, being followed up with the diagnosis of bronchiectasis, asthma, and scoliosis, had been using inhaled corticosteroid/long-acting beta-2 agonist. The patient did not smoke or drink alcohol, yet her oral hygiene was poor. When she applied to the emergency department due to fever, cough, and increased amount of sputum, her pulse O2 saturation level was 86% (in room air), arterial blood pressure was 118/62 mm Hg, pulse was 86 beats/min, and the temperature was 37.5 °C. On physical examination, there were coarse rales in the left lower zone of the lung. In laboratory tests, pathological values consisted of a C-reactive protein (CRP) of 325 mg/L, a leukocyte count of 25.84 K/uL, and a neutrophil ratio of 95.8%. In the chest computed tomography (CT) imaging, a bronchiectasis image was observed in the middle-lower zone of the left lung (Fig. 1a). Parenteral moxifloxacin treatment was initiated for the patient who was hospitalized. Growth of Pseudomonas aeruginosa was detected in sputum culture, and piperacillin-tazobactam was added to the treatment. In the follow-up, since there was no fever response, increased sputum purulence and amount, decreased respiratory sounds on the left in physical examination, and progression in CRP levels, the patient had another chest X-ray, and total density increase was observed on the left hemithorax (Fig. 1b). Chest CT revealed pleural effusion on the left side. With tube thoracostomy, foul-smelling purulent fluid was drained (Fig. 1d). The biochemistry evaluation of the pleural fluid was compatible with empyema (Table 1). Actinomycosis was diagnosed through pathological examination after dense neutrophils were observed in pleural fluid cytology, sulfur granules in Hematoxylin–Eosin staining (Fig. 2a) and hyphae structures in Giemsa staining (Fig. 2b). There was no growth in the pleural fluid culture. SARS-CoV2 RT-PCR test was also positive in nasopharyngeal sampling. Ampicillin-sulbactam treatment was started. On the 10th day of the tube thoracostomy, the chest tube was removed due to decreased fluid and the radiological response. At discharge, the treatment was continued as amoxicillin + clavulanic acid 2 g/day. At the 2nd-month follow-up, symptoms decreased, and a significant regression was observed in the chest X-ray (Fig. 1c). The patient's treatment still continues.

**Table 1:** Biochemistry test values of pleural fluid and blood serum

	**P**leural fluid	Blood serum
Lactate dehydrogenase	** > 7500 U/L**	371 U/L
Protein	4.7 g/dL	62 g/L
Glucose	**8 mg/dL**	110 mg/dL
Albumin	2.4 g/dL	30 g/L

## Discussion

Predisposing factors in actinomycosis infection of endogenous origin include alcoholism, poor oral hygiene, gingival diseases, suppression of the immune system, long-term use of intrauterine devices, and chronic structural respiratory system diseases such as bronchiectasis [1, 2]. Our case was diagnosed with asthma and bronchiectasis and had poor oral hygiene.

Actinomycosis cases usually present with the appearance of consolidation, mass, cavity, or abscess radiologically [3], and pleural effusion rarely develops. In Actinomyces infections, Acinobacillus actinomycetesmcomitans, Eikenella corrodens, Klebsiella spp, Enterobacteriaceae, Fusobacterium spp, Bacteroides spp, Capnocytophagia spp, Staphylococci spp, and Streptococci spp have been isolated, but their role in the pathogenesis remains unclear [4–9]. Oxygen deprivation due to polymicrobial infection is likely to increase, facilitating the occurrence of actinomyces infection [4, 6].

In aerobic-based microbial cultures of patients with bronchiectasis, Haemophilus influenza (14–47%), Pseudomonas aeruginosa (5–31%), and Streptococcus pneumonia (2–14%) were reported to be the most frequently isolated pathogens [10–13]. Although not as much as aerobic agents, Veillonella, Prevotella, and Actinomyces can also be reproduced from anaerobes. Nicotra et al. detected anaerobic organisms in 1.6% of cases with bronchiectasis (10). In healthy non-smokers, the lower respiratory tract is considered sterile. Colonization with potentially pathogenic microorganisms often develops in the presence of chronic bronchitis, chronic obstructive pulmonary disease (COPD), bronchiectasis, bronchial obstruction, and tracheostomy, in which oropharyngeal protection is compromised [14]. Colonization frequency can reach up to the rates of 60–80%. Thus, this condition creates a risk factor for lung infections and causes increased secretion of inflammatory mediators, progressive tissue damage, and increased airway obstruction [15, 16]. Among the colonizing microorganisms, Pseudomonas aeruginosa has a critical place. Pasteur et al. determined P. aeruginosa colonization in bronchiectasis at a rate of 24% [11]. In our case, P. aeruginosa growth was detected in the sputum, which we considered as colonization, and SARS-CoV2 RT-PCR was positive in the nasopharyngeal sampling. In the literature, cases of rhinosinusitis and osteomyelitis due to actinomycosis in the post-COVID period have been reported [17–19]. However, our case was the first in the literature due to the coexistence of COVID-19, Pseudomonas, and thoracic actinomycosis. It is also a warning to physicians regarding similar cases that may occur during the COVID-19 pandemic.

Gram staining and histopathological evaluation are more sensitive than culture in diagnosing actinomycosis. The reason for this situation is the slow reproduction of Actinomyces species in an anaerobic culture medium. In addition, previous empirical antibiotic therapy reduces the feasibility of cultures [4, 20].

In our case, there was no growth in the pleural fluid culture. The diagnosis was made by the presence of sulfur granules and hyphae structures in cytology.

Although the treatment regimen and duration are unclear, beta-lactams such as penicillin G, amoxicillin, ceftriaxone, doxycycline, and clindamycin, along with erythromycin for pregnant women, are recommended. The duration of treatment is recommended as 6–12 months [1]. Surgery is especially useful in the presence of an abscess, empyema, and massive hemoptysis (5,6). In our patient, good treatment response was observed in the 2nd-month to oral amoxicillin-clavulanic acid treatment, and the patient's treatment still continues.

## Conclusion

Polymicrobial infection factors should be considered in patients with chronic structural lung diseases. It is crucial to perform a cytological examination of the materials in the absence of growth in cultures and include diseases such as actinomycosis in the differential diagnosis.

## Data Availability

Not applicable.

## Abbreviations

COVID-19: Coronavirus disease 2019
SARS-CoV-2 RT-PCR: Severe Acute Respiratory Syndrome Coronavirus- 2 Reverse Transcriptase Polymerase Chain Reaction
CRP: C-reactive protein
CT: Computed tomography
COPD: Chronic obstructive pulmonary disease
H&E: Hematoxylin–Eosin
